# Patient perspectives about the healthcare of chronic musculoskeletal pain: Three patient cases

**DOI:** 10.4102/ajod.v5i1.216

**Published:** 2016-05-18

**Authors:** Dawn Ernstzen, Quinette Louw, Susan Hillier

**Affiliations:** 1 Division Physiotherapy, Stellenbosch University, South Africa; 2 International Centre for Allied Health Evidence, School of Health Sciences, University of South Australia, Australia

## Abstract

**Background:**

Consideration of the patient’s perspective in healthcare is important because it may inform holistic and contextually relevant management strategies.

**Objectives:**

The purpose of this study was to explore patients’ experiences and perspectives about their chronic musculoskeletal (CMSK) pain and its management in the private healthcare sector in South Africa. This work was done as a pilot study to test, adapt and finalize an interview schedule.

**Methods:**

A descriptive, qualitative study was conducted. The sampling was purposive. Three patients with CMSK pain were recruited to participate in in-depth individual interviews. The interviews were recorded and transcribed ensuring confidentiality. Inductive, thematic content analyses of the transcripts were undertaken. Initial codes were assigned and a code book developed, which was applied to the transcripts to develop categories and themes.

**Results:**

Four themes emerged from the data: (1) the participants sought understanding about the pain’s origin and the reason for pain persistence; (2) pain impacted their lives in multiple ways; (3) the participants depended on healthcare providers (HCP) for guidance and support; and (4) they had the option of acceptance of chronic pain.

**Conclusion:**

The participants’ knowledge about their health condition had important implications as it influenced their perspectives on pain and its management. The pain presented the participants with several challenges, which included developing an understanding about pain and coping with the impact of pain in their lives. HCPs were perceived to play an important role in empowering or disempowering the participants.

## Introduction

Chronic musculoskeletal (CMSK) pain and its management present a challenge to patients, healthcare providers (HCPs) and communities. The condition is classified as part of chronic non-malignant pain, which includes musculoskeletal, neuropathic visceral pain and pain from sickle cell disease (World Health Organization [WHO] 2007). CMSK pain comprises of pain associated with joints, muscles, tendons and nerves that persists for longer than 12 weeks, and thus beyond the expected healing time (Blyth *et al*. 2001). It has been recognised as a global healthcare concern and affects many societies, including sub-Saharan Africa, where CMSK is a major cause of disability and morbidity (WHO 2003; Furlan, Reardon & Weppler 2010; Rauf *et al*. 2014). The condition has a significant impact on physical and psychological health and functions, participation in life roles, and thus ultimately on the quality of life of the individual (Foster *et al*. 2003; Furlan *et al*. 2010).

Foster *et al*. (2003:402) call for a patient-centred approach to the problem of CMSK pain, to ‘illuminate the long neglected patient’s perception of their problem and its management; and thus the dynamic interaction between the condition, the patient’s perception and the practitioner’s influence’. Individuals with chronic pain often perceive their condition to be neglected (Upshur, Bacigalupe & Luckmann 2010), therefore a patient-centred approach is essential. Understanding the patient as a person and the individual experience of illness is a core aspect of a patient-centred approach (Mead & Bower 2000). Kidd, Bond & Bell (2011) describe patient-centred healthcare as the patient being central within the consulting relationship, resulting in understanding from the patient’s perspective, which may ultimately influence healthcare utilisation (Wagner *et al*. 2005). Furthermore, understanding the patient’s perspectives may inform management strategies that are contextually relevant and acceptable to the patient, thus providing for a holistic and patient-centred management plan as a part of quality healthcare.

Several studies on patient perspectives about the healthcare management of chronic pain in well-established healthcare systems have been conducted internationally. The findings of these studies focus on patients’ understanding of their pain, their perspectives regarding treatment received, barriers and facilitators to care, patient–provider relationships and patient-centredness (Allegrettia *et al*. 2010; Kidd *et al*. 2011; Potter, Gordon & Hamer 2003; Upshur *et al*. 2010). Little information is available on the process of coming to terms with chronic pain. Skuladottir & Halldorsdottir (2008) addressed this gap in the literature by developing a theory on women’s processes of making sense of chronic pain. In their theory, women are challenged to cope with pain, to live with pain and to find meaning in their suffering. According to this theory, HCPs are seen as playing a powerful role assisting women in regaining control and sense in their lives.

Studies on patient perspectives regarding chronic pain care in Africa are scarce. One study in South Africa investigated the satisfaction of patients with a chronic pain management group (Parker *et al*. 2009). The current study builds on the knowledge base on patient perspectives and experiences in the South African (SA) healthcare context. The SA healthcare context is characterised by a private as well as a public healthcare system (Rowe & Moodley 2013). This study focuses on private healthcare. This work is an essential start to identifying the contextual factors that might impact patient perspectives and healthcare delivery.

## Objectives

The objective of this study was to explore patients’ experiences and perspectives of their CMSK pain and its management in the private healthcare sector in South Africa. This pilot study was done to test, adapt and finalise an interview schedule for a larger study on clinical guidelines for the management of CMSK pain.

## Research method and design

### Study design

A descriptive, qualitative study was conducted, using an interpretive research paradigm, to study the lived experience of chronic pain. Britten (2006) postulates that qualitative research investigations focus on individuals in their natural setting and are concerned with the participants’ perception of their world. In this study, semi-structured individual interviews were conducted to discover the participants’ experiences, understanding and framework regarding their CMSK pain and its management.

### Study setting

The study was conducted in the Western Cape, South Africa, and involved patients who received healthcare in the private healthcare sector.

### Sampling

Purposive (strategic) sampling in the form of criterion sampling was used (Palys 2008). The key criteria linked to the study objectives included that the participants had to have chronic pain (constant pain for longer than three months) and the pain had to be musculoskeletal in origin. Persons with chronic pain of non-musculoskeletal origin, for example, cancer pain, neuropathic pain and chronic pain from sickle cell anaemia, were not eligible to participate. This exclusion criterion was set as these types of chronic pain have different pathological processes, clinical symptoms and management processes, which could lead to different patient perspectives. Male or female adults were eligible to participate. Furthermore, eligible participants should have received healthcare for their condition in the SA private healthcare setting. Starks & Trinidad (2007) advise that a typical sample size for a phenomenological study should range from 1–10 participants to identify the core element of the phenomenon. For the purpose of this pilot study, three patients with CMSK pain were recruited from three healthcare practices (one patient per practice).

### Instrumentation

In-depth individual interviews were conducted, because of the possibly sensitive nature of the information. The principal investigator (PI) developed an interview schedule (Box 1) based on similar research, and its content was evaluated by the co-researchers and by two external auditors who were familiar with the research objectives. The interview schedule was designed to elicit the participants’ narrative and perspectives regarding their pain and consequential healthcare management.

BOX 1 Main interview schedule.

1. Please tell me your story about your pain, from when it started up until today.
  Injury source?
  Pain becoming persistent – possible reasons?
2. Please describe to me how the pain influences your life.
  Functional ability, social and family participation
3. Could you tell me about the treatment you received for your pain?
  Healthcare providers involved
  Patient journey or pathway of care
  Satisfaction/dissatisfaction with treatment options
4. Which aspects of treatment helped you the most?
5. In your view, what can be done to optimise/improve the treatment of your pain?
  Key messages to healthcare providers to optimise –
  Pain management characteristics
  Individual characteristics
  Treatment characteristics
  Healthcare system characteristics
  Community or context characteristics
  Expectations regarding treatment

### Research procedures

HCPs, which assessed patients, were requested to identify eligible patients, inform them about the study and ask permission to refer them to the PI. The PI contacted the eligible patients and arranged to conduct the interviews in the participants’ home or work setting to allow for a natural milieu, as advocated by Britten (2006). Prior to the interview, the PI explained the purpose of the research. Informed consent was obtained, and the participants completed a short questionnaire to provide their socio-demographic information. Each interview lasted approximately 30 min – 40 min. The interviews were conducted in Afrikaans, which was the home language of the participants. Interviews were recorded on a digital voice recorder, then downloaded to the PI’s personal computer and allocated a unique serial number.

The positionality of the researcher forms an integral part of the research process. The personal characteristics of the interviewer (age, occupation, gender) can influence the data collection and analysis (Karnieli–Miller, Strier & Pessach 2009). The PI was a female physiotherapist who did not have chronic pain but has been involved in the healthcare of patients with CMSK pain. The participants knew that the researcher was a physiotherapist, and this aspect could have influenced the participants’ responses about HCPs. The researcher has worked in private and public healthcare settings, including at the primary and tertiary healthcare levels, and has experience in the academic setting. This background could have influenced her interpretation of the data. The participants as well as the researcher were female, and this aspect could have eased communication, resulting in openness and approachability. The researcher has experience in qualitative research and has undergone training in qualitative methods, including interviewing, to prepare for the data collection and analysis.

## Analysis

The interviews were transcribed verbatim by the PI. Inductive, thematic content analysis of the interview transcripts were done, as described in Pope, Ziebland and Mays (2006). Data analysis involved an iterative process of immersion in the data, familiarising oneself with the data, highlighting significant statements (quotes), creating a code book, coding the data, developing clusters of meaning (categories), establishing themes and, finally, interpretation and validation of the data. The PI independently assigned initial codes, then revisited the data to check accuracy as part of validity checking. The initial analysis was done using the Afrikaans texts. The quotes were translated by the PI to enable external auditing. The two external auditors, who were familiar with the research objectives and the interview schedule, evaluated the data coding of two transcripts as part of validation. The external auditors provided comments on the accuracy of the categories assigned to the quotes and the themes that arose from the categories. The co-researchers approved the final themes. A summary of the findings was communicated to the participants to aid validation.

## Ethical considerations

The study protocol was approved by the Health Research Ethics Committee of Stellenbosch University, South Africa (S14/01/018). Informed consent was obtained from participants. Participation was voluntary, and the participant could withdraw from the study at any point. The participants’ personal information was kept confidential. It is acknowledged that qualitative research involves power relations (Karnieli–Miller *et al*. 2009). The researcher and the participant are involved in the process of power sharing, which entails continuous establishing of boundaries and negotiation of power. The researcher thus requested the participants’ consent to participate in the interview. The interviews were done in the participants’ natural setting (home or work), which was an unfamiliar setting for the researcher. At the start of the interview, the researcher built rapport by creating an atmosphere of trust, emphasising that the researcher wanted to learn from the participant by listening to the participant’s story. The researcher thus acknowledged the participant’s contribution.

## Results

### Description of participants

Three female patients with CMSK pain participated in the study. All three were married and had two or three adult children. Pseudonyms will be used to discuss the participants’ responses. Anne and Sarah had obtained a tertiary education and were working full-time in management positions. Delia had previously worked as a manager in retail but was unemployed at the time of the interview. Anne had chronic bilateral posterior lower limb pain for 15 months with no specific precipitating event. She rated her average pain as 6/10 on the Visual Analogue Scale (VAS). Sarah had chronic widespread pain, which started about two years earlier without any precipitating event. She rated her average pain as 4/10 on the VAS. Delia had chronic low back pain and leg pain for 15 months, after she sustained an injury at work. She had a lumbar fusion one year after the incident. She rated her average pain as 7–8/10 on the VAS.

### Main findings

Four major themes emerged from the data, the (1) search for understanding, (2) impact of pain, (3) role of the healthcare provider, and (4) acceptance of pain.

Below, the data are described in their context according to each theme, followed by substantiating quotes. All quotes have been translated from Afrikaans to English by the PI for the purpose of this article.

### The search for understanding

Although the pain features of Anne, Sarah and Delia were different, their narratives were strongly focussed on their pursuit of understanding the origin of the pain and the reason for its persistence. Understanding pain was important to them to make sense of the pain and to complete their pain puzzle.

‘It is not as if I am fabricating [*sic*] the pain. … I live with it everyday. The pain can’t come from out of nowhere; it has to originate somewhere’. (Anne)

‘You are uncertain … and you keep searching, and you start wondering’. (Sarah)

Delia’s narrative focussed less on the search for the source of the pain and more on the reasons for its persistence. She started blaming herself for the persistent pain:

‘I think it is because I am an impatient person. I don’t give this thing time to heal. I must stick to the rules’. (Delia)

Anne consulted several HCPs in her search for the source of her pain. This process left her feeling despondent:

‘No one really has an answer. You go back to that person, but he cannot find anything faulty in the area that he tests. Next time you go to a different person. Later, you ask yourself: What is wrong with me?’ (Anne)

The above quote also represents the participants’ experience of a lack of communication and collaboration between HCPs, which hindered continuity of care and the understanding of her condition. These perspectives about the lack of collaboration between HCPs were authenticated when analysing the care pathway that the participants followed. The participants were referred to and consulted several HCPs through an inconsistent healthcare pathway. This disconnected care pathway further strengthened Sarah’s fears and uncertainty about the origin of her pain:

‘They send you around, and later they also don’t know where-to next. The medication is not effective. What should you do next? What do you do now?’ (Sarah).

In seeking for understanding, Anne and Delia also consulted the Internet for information on their condition.

## The impact of pain

### Emotional impact

Fear, worry and uncertainty about the pain, its origin, and its effects on their lives were expressed as dominant emotions during the process of finding answers for the pain. The pain affected their very being, which was reflected in the following statements:

‘And then the uncertainty … maybe it [*the pain*] is going to persist. … Is this your future or what?’ (Sarah)

‘In my head, I wondered if it is cancer or something and nobody picks it up’. (Anne)

‘Now I am very worried – what went wrong? I really cannot face another operation. I told my husband, this pain is driving me crazy, from one day to the other …’ (Delia)

Delia kept a diary in which she recorded the behaviour of her pain, which emphasises her vigilance about finding a pain pattern:

‘You need to monitor yourself – make notes of what you do, and then you can find out: What did I do yesterday that makes me feel this way today?’ (Delia)

### Functional impact

Pain interfered with the participants’ functional abilities, including self-care, work and limited participation in leisure activities. The unpredictability of the occurrence of pain was a particular concern for Sarah:

‘There were times I decided to go for a slow stroll, but then everything got worse. And you can’t go on. I used to like going for hikes on the mountain; now I can’t do what I used to do, I can’t do easy walks’. (Sarah)

‘I could not sit, I could not work. I walked with great difficulty. It just got worse’. (Sarah)

‘The thing that restricts me the most is that I can’t be active as a result of the pain. I can’t be myself’. (Anne)

‘I cannot be disabled? I walk from the car to the entrance of the mall, and then I am tired. Not only tired, but also sore on top of it. You know, the pain restricts me a lot. I can’t use public transport’. (Delia)

‘Now the pain is not there, and then suddenly, tomorrow or the day after, the pain is back!’ (Delia)

Delia also lost her job after the incident; however, she chooses to focus on the positive aspects:

‘And my employer decided that they do not need my services any more. I was unfortunate to be dismissed from work, but I am fortunate that I can be at home. I am not going to stress about work. We work together as a family’. (Delia)

### Impact on family

The participants were thankful about the support they received from their family. However, only Delia commented on the impact of her suffering on her family:

‘The drama that I put my family through! … Yes, they are my support system; my husband is wonderful’. (Delia)

## The role of the healthcare provider

The search for understanding pain, as well as the immense impact of pain, left the participants vulnerable. Each participant acknowledged that she turned to HCPs for guidance, care and support. Anne appreciated the support she received from her HCP:

‘My doctor went through a lot of trouble for me, to go through all the elements and to eliminate that which is not causing the pain. She phoned the specialist and got the information for me’. (Anne)

However, Sarah was referred to a specialist and had to wait three months. She felt that more could have been done to support her during this time of uncertainty.

‘What would have been good for me is not only to refer me to the specialist, and not be worried that I can only get an appointment in three months’ time, but rather to help me to decide what I should do in the meantime, while I have to wait …’ (Sarah)

Sarah later identified the characteristics of an HCP who supported her:

‘And it is good for me. You feel you can talk to him. He does not let you feel that you are asking stupid questions. It is important, because you are unsure. The way he approaches it, is good and important’. (Sarah)

Delia also mentioned aspects that she valued during her journey:

‘They were good; although they did not really understand the pain, they were sympathetic. They showed empathy, and they listened’. (Delia)

The participants valued a collaborative relationship between the patient and the HCP and expressed a desire to be part of the solution. The collaborative relationship was described as open communication between the patient and the HCP and approachability of the HCP.

‘What is good about Dr X is that he consults me and provides me with thorough information about decisions’. (Sarah)

The participants mentioned several attributes of the HCP that fostered patient-centred care and positively influenced the participants’ coping mechanisms. These attributes included approachability, good communication skills, a caring nature, genuineness, trustworthiness and guidance.

However, some features of HCPs frustrated the participants, and this formed a barrier to care. Particular instances mentioned by the participants included a lack of understanding of the pain, the HCP’s not believing that the patient’s pain was real, and statements that the pain was in the patient’s head:

‘Nobody understands your pain, hey. They would not know what you are talking about’. (Delia)

‘They told me that I must get my head right, that I must manage the pain in my head, but the pain is not in my head! They must try and understand that the pain is not in your head. It is not the origin of the pain. Stop saying to the patients to get your head right – it is very frustrating to hear that’. (Delia)

## The acceptance of pain

Sarah started a process of accepting pain as a constant companion in her life when her condition was diagnosed, and she thus found a credible explanation for the pain’s persistence. Once she received this explanation, she felt as if her life could continue:

‘Also, the fact that you can now put a name to the condition … you are not fabricating the pain’. (Sarah)

‘I had to adapt and realize there are certain things that I cannot do. The fact that I walk in the mountains again is good for me. And yes, I accepted long ago that I cannot do now what I did earlier. That is it. Other people struggle with worse things. You learn to manage it’. (Sarah)

However, Anne, at the time of the interview, had not yet found a credible explanation for her persistent pain. She remained actively seeking an answer for the pain, and her quest to complete the puzzle of her pain continues:

‘I feel satisfied with all the tests done, but I am dissatisfied that I still don’t have an answer. I can’t pinpoint it [*the pain’s origin*]’. (Anne)

Delia also remains hopeful for an improvement:

‘But it will never go completely away, but he said the pain would get about 80% better, and that is what I would want. It is never going to be the same again’. (Delia)

## Discussion

The primary aim of this study was to discover patients’ experiences and perspectives regarding the healthcare management of their CMSK pain. The findings indicate that the participants were faced with several challenges, as identified in their narratives about their pain. The first challenge was seeking to understand the origin of the pain, as well as the reasons for its persistence. The pain left the participants vulnerable and dependent on the guidance and support from HCPs. The next challenge was the participants having to come to terms with the immense impact of chronic pain on their lives. And finally, there was the challenge of accepting pain as a part of their lives.

The participants reported the dominant emotions of fear, anxiety and worry accompanying their realisation of the significant impact of pain in their lives. Under these circumstances, the participants relied not only on family support but also on support and guidance from HCPs. It could be deduced that there is a strong impetus for HCPs to address patients’ concerns about their pain. The participants were actively seeking information and reassurance about their condition. Hayes & Hodson (2011) advocate early educational interventions for people in pain, to assist patients in making informed choices about the pain and limit its effects. The notion relates to education as therapy. One such educational approach, namely pain neurophysiology education (PNE), was found to be beneficial to aiding the patient’s understanding of CMSK pain (Clarke, Ryan & Martin 2011; Louw *et al*. 2011). PNE focusses on explaining the biology of nociception and the reasons for pain becoming persistent, to enable patients to re-conceptualise their pain experience. When patients understand their pain experience, it might alleviate their worries about the pain and in return decrease the disability associated with chronic pain.

The findings indicate that HCPs may play a central role in the patient’s journey towards understanding and acceptance of pain. This is congruent with the theory of Skuladottir & Halldorsdottir (2008) that HCPs play a powerful role in empowering or disempowering the patient. The participants identified several elements that were important to them in the healthcare management process and can be seen as empowering. These elements include a collaborative relationship between the patient and the HCP, where communication, approachability, empathy and trust are central. This collaboration is necessary to achieve shared decision making, empowerment and a therapeutic alliance between the patient and the HCP. Upshur *et al*. (2010) contend that the attributes of the HCP play an important role in forming a therapeutic alliance, to enhance the patient–provider relationship, and contribute to patient satisfaction with chronic pain care. However, disempowering elements were also noted, such as HCPs not believing or understanding the participants’ pain experience. Egeli *et al*. (2008) also noted that patients with chronic pain could be disempowered by HCPs. One participant was particularly disempowered by comments that the pain was in her head, whereas the patient was convinced that the pain was in her back. This may be an example of the negative effect that inadequate communication has on the patient–practitioner relationship. It is thus important that HCPs be attentive to any form of miscommunication between them and their patients, which could be detrimental to patients’ coping ability.

The participants identified two healthcare system factors that acted as barriers to optimal pain care, namely the lack of communication and collaboration between different HCPs, which in turn led to the second system factor, a disconnected care pathway in private practice. The participants became discouraged as they were depending on guidance from the HCP but often had to make their own decisions about their pathway of care. These system factors could be addressed by interdisciplinary care through a team approach. Hayes & Hodson (2011) also identified a lack of collaborative practice in pain care in a healthcare setting in Australia, and they reported on several changes they made to develop a systems approach for chronic pain care. Hayes and Hodson (2011) and Wagner *et al*. (2005) advocate a systems approach that focusses on patient-centred and interdisciplinary care as necessary to address chronic pain. There are different factors that may play a role in establishing such a patient-centred and interdisciplinary system in the SA private healthcare sector. They include interdisciplinary versus solo practices, information technology options to improve communication, cost, patient advocates and patient preferences. A thorough analysis of the barriers and facilitators is needed to determine how different factors would enable interdisciplinary care. The SA healthcare system is currently a system in transition (Rowe & Moodley 2013). The introduction of the National Health Insurance could play an important role in establishing a systems approach that emphasises interdisciplinary collaboration.

The participants in this study went beyond a narrative of their pain to introduce the concept of acceptance of pain. Their willingness to accept pain was related to their understanding of the basis of their pain, whilst their coping strategies were positively influenced by empowerment by HCPs. Acceptance of chronic pain has been defined as living with pain without attempts to reduce or avoid it; engaging in functions and daily activity regardless of the pain and the willingness to continue with enjoyable activities despite having pain (McCracken & Eccleston 2003). Acceptance of pain thus requires an active approach. Preliminary evidence from descriptive studies indicates that acceptance-based rehabilitation may lead to positive results in pain, disability, depression, anxiety and quality of life (McCracken & Eccleston 2003; McCracken, Vowles & Eccleston 2005).

This study, albeit small and non-generalisable, raises several thoughts and questions that could be further investigated. These include the several challenges that patients face in their personal journey of realisation to acceptance of CMSK pain. The study expands on patient expectations about healthcare management of CMSK pain and emphasises the powerful role of the HCP as a source of support and guidance. The participants had definite and strong expectations about their healthcare management, which is congruent with the notion of patient-centeredness as described by Mead & Bower (2000). The findings strengthen the need for interdisciplinary collaboration to effectively address chronic pain. As a result of this pilot study, we adapted our interview schedule to include more specific probing questions regarding contextual factors as well as coping strategies, specifically relating to Question 3 and Question 5 (Box 1).

In our search for uniquely contextual elements in the SA context, only two aspects could be identified. The first is the participants’ experience of a lack of collaboration between different HCPs in private practice. The second contextual factor concerns contextualisation of educational interventions to ensure that it is appropriate en relevant to the patient and to optimise communication between the patient and the HCP.

## Limitations

There are several limitations that need to be taken into account when interpreting the results of this pilot study. The sample size is small, and the lived experiences of three female participants with CMSK pain are investigated in this study. The findings of the study cannot be generalised as a more diverse sample might provide a broader range of perspectives. As a next step in the research process, more patients in different healthcare contexts need to be interviewed to obtain theoretical data saturation. Furthermore, it is acknowledged that the personal characteristics of the interviewer (age, occupation, gender) might have influenced interviewee responses. The researcher is a physiotherapist and thus could have influenced the participant responses toward HCPs. However, the open responses and feedback provided by the participants suggest that this was not a barrier to them sharing their experiences. The interviews and initial analysis were done in Afrikaans and later translated to English by the PI. This translation might have impacted the richness of the quotations.

## Conclusion

The narratives of the three participants with CMSK pain indicate that their journey with chronic pain presented several challenges. The participants were actively seeking an understanding about the source of pain and the reason for its persistence in order to make sense of the pain. A disconnected healthcare pathway was barrier to understanding pain. HCPs played an important role in empowering or disempowering the participants in their journey with chronic pain. Addressing the above factors may enhance the quality of care for patients with CMSK pain.
